# Understanding zoonotic pathogens and risk factors from wildlife in Southeast Asia: a systematic literature review

**DOI:** 10.1080/01652176.2025.2475990

**Published:** 2025-03-10

**Authors:** Ha Thi Thanh Nguyen, Johanna F Lindahl, Bernard Bett, Hung Nguyen-Viet, Steven Lâm, Thang Nguyen-Tien, Fred Unger, Sinh Dang-Xuan, Thanh Xuan Bui, Hien Thanh Le, Åke Lundkvist, Jiaxin Ling, Hu Suk Lee

**Affiliations:** a Department of Medical Biochemistry and Microbiology, Uppsala University, Uppsala, Sweden; b International Livestock Research Institute, Hanoi, Vietnam; c International Livestock Research Institute, Nairobi, Kenya; d Swedish Veterinary Agency, Uppsala, Sweden; e Ho Chi Minh City Department of Health, Ho Chi Minh Center for Diseases Control, Ho Chi Minh, Vietnam; f Ho Chi Minh City University of Agriculture and Forestry, Ho Chi Minh, Vietnam; g College of Veterinary Medicine, Chungnam National University, Daejeon, Republic of Korea

**Keywords:** Zoonotic diseases, wildlife, Southeast Asia, risk factors, one health

## Abstract

The COVID-19 pandemic has demonstrated the significance of the human-animal interface in the emergence of zoonotic diseases, with wildlife serving as an important source of infection. A better understanding of the specific pathogens and mechanisms involved is vital to prepare against future outbreaks, especially in Southeast Asia, a hotspot for zoonotic diseases. This paper reviews the published literature on wildlife zoonoses in this region from 2012 to 2022. The results show a diverse range of potential zoonotic pathogens and the widespread occurrence of zoonotic diseases from wildlife. Drivers of zoonotic pathogen spillover include (i) environmental factors (e.g. animal habitat disruption, environmental conditions, exposure to contaminated water/food/soil), (ii) animal factors (e.g. movement patterns, age-related susceptibility), (iii) human factors (e.g. lack of awareness, poor hygiene practices, age, gender and income) and (iv) human-animal-environmental interface factors (e.g. close contact between humans and animals, exposure through visiting animals and presence of vectors). The diverse drivers of zoonoses in Southeast Asia put its communities at risk for infection. To mitigate these risks, global health efforts should consider adopting a One Health approach to foster collaboration across human, animal, and wildlife health sectors. This could involve educating communities on safe animal interactions and improving disease surveillance.

## 1. Introduction

Coronavirus disease 2019 (COVID-19) has placed a spotlight on the threats posed by zoonoses – diseases that can be transmitted between animals and humans – in Southeast Asia, where the interface between animals and humans is particularly pronounced. Southeast Asia’s intensive livestock production systems, rapid urbanization, extensive domestic and cross-border trade in animals and their products, poor farm biosecurity practices, presence of wet markets, and diverse farming practices – including wildlife farming, farming of wild, captive, and undomesticated animals – make it a hotspot for emerging and re-emerging zoonoses (Chongsuvivatwong et al. 2011; Wacharapluesadee et al. 2021). The burden on public health is considerable, with researchers estimating that the top 56 zoonotic diseases are responsible for 2.7 million fatalities annually worldwide (Gebreyes et al. 2014).

Over 60% of emerging infectious disease events are caused by zoonotic pathogens, with the majority coming from wildlife (Jones et al. 2008). Examples include the Ebola virus (Bausch et al. 2007), avian influenza viruses (Kalthoff et al. 2010; Adlhoch et al. 2023), severe acute respiratory syndrome coronavirus (SARS-CoV) (Wang et al. 2018), Middle East respiratory syndrome coronavirus (MERS-CoV) (Omrani et al. 2015), and more recently, the SARS-CoV-2 pandemic, which began in 2019 (Delahay et al. 2021). These diseases have either directly spilled over into human populations or have been transmitted *via* intermediaries including livestock. While there has been growing scientific interest in zoonoses originating from wildlife, the approach to managing outbreaks typically remains reactive. Emphasis is placed on developing vaccines and drugs rather than preventing the disease emergence and spillover events (Hilderink and de Winter 2021).

In Southeast Asia, the expansion of wildlife farming, marketing, and consumption systems increases the risk of zoonotic disease spillover. Indeed, the wildlife trade has emerged as a key source of zoonotic disease outbreaks in the region (Taylor et al. 2001; H. Li et al. 2021). Furthermore, this wildlife trade – characterized by complexity, unsustainability, and lack of regulation enforcement – facilitates the movement of infected animals and their products across borders, elevating the likelihood of introducing zoonotic pathogens on an international scale (Sarma 2017). While wildlife farming can enhance economic opportunities and provide an alternative food source for people who rely on them for their livelihoods (TRAFFIC 2008; Huong et al. 2020), the benefits of wildlife trade must be balanced against risks. Implementing preventative measures that respond to community needs is essential to mitigate these risks.

Considering the health burden of zoonoses, the key role of wildlife in transmitting zoonoses in Southeast Asia, and the potential for wildlife zoonoses to transcend borders, our systematic literature review aimed to understand the range of wildlife zoonotic pathogens and transmission pathways in the region. Specifically, our objectives were to review and synthesize the published literature on wildlife zoonoses in Southeast Asia. This review helps to inform prevention efforts to reduce the risks of wildlife zoonoses, not only in Southeast Asia but also globally.

## 2. Methods

We examined the published literature using a systematic literature review methodology - Preferred Reporting Items for Systematic reviews and Meta-Analyses (PRISMA) involving a multi-stage process of search, selection, extraction, and synthesis of the literature (Page et al. 2021).

### 2.1. Search strategy

We searched for publications using three databases: PubMed, ScienceDirect, and Web of Science. We used the following set of keywords (with slight differences in the search terms to match how each platform works):

- PubMed: (wildlife OR wild animal*) AND (Brunei OR Cambodia OR Indonesia OR Lao PDR OR Malaysia OR Myanmar OR Philippines OR Singapore OR Thailand OR Timor-Leste OR Vietnam OR Southeast Asia) AND (zoono*)
-  ScienceDirect: (wildlife OR wild animal) AND (Southeast Asia) AND (zoonoses)
- Web of Science: ((zoono*) AND (wildlife OR wild animal*) AND (Brunei OR Cambodia OR Indonesia OR Lao PDR OR Malaysia OR Myanmar OR Philippines OR Singapore OR Thailand OR Timor-Leste OR Vietnam OR Southeast Asia))

We imported retrieved records into Endnote (version X9) to store and remove duplicates. We also hand-searched the reference lists of all included studies to identify relevant studies not captured in the searches.

### 2.2. Relevance screening and eligibility

A two-step relevance screening strategy was employed by three independent reviewers (HN, PH and AY). First, the titles and abstracts of publications were screened; next, all publications deemed potentially relevant went through a review of the full text. Publications were considered to be relevant if: they included studies that were published between January 2012 and November 2022; and, they described any aspect of wildlife zoonoses in the context of Southeast Asia. The three reviewers met regularly throughout the screening process to resolve disagreements and discuss any uncertainties related to study selection.

### 2.3. Data extraction and synthesis

We created a data charting form to extract data from the studies. We captured the general characteristics of the article, including authorship details, title, and year of publication. Furthermore, we extracted data relative to our review objective, including the name of pathogens, risk factors (i.e. environment, human, animal, or human-animal-environmental interface factors), animal species, host class, host order, study area, source, sample type, sample size, number of positive samples, diagnostic method, prevalence, and 95% confidence interval (CI). For pathogens that were reported from multiple studies, we presented the finding with the higher percentage of prevalence. If the papers did not have information on prevalence or 95% CI, we calculated these based on the data presented in the papers. Results were exported into Excel (Microsoft version 2016) for descriptive analysis.

## 3. Results

### 3.1. Overview of relevant publications

**Figure 1.**
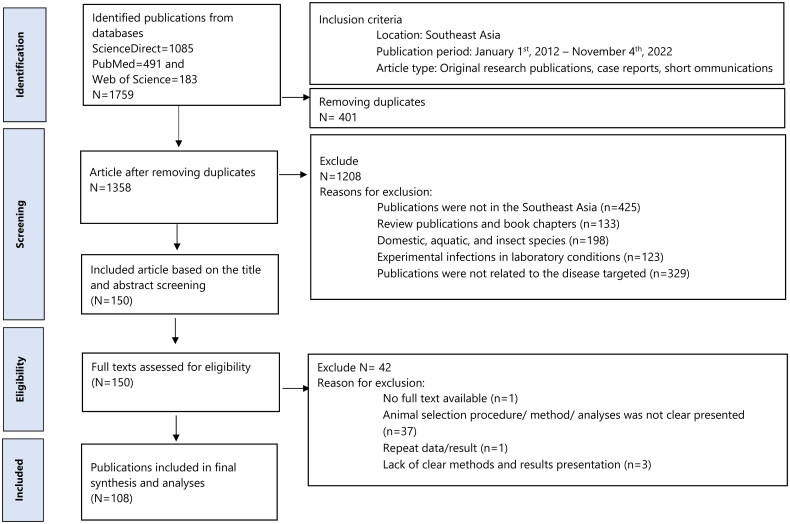
Schematic flow diagram of the literature selection for the review (www.prisma-statement.org).

A total of 1,759 publications were identified through the database search. After removal of duplicates and non-relevant publications, a total of 108 publications were included in the final synthesis (Figure 1). A list of all included publications and article characteristics is presented in Supplementary Table S1.

### 3.2. Geographical and temporal distribution of publications

**Figure 2.**
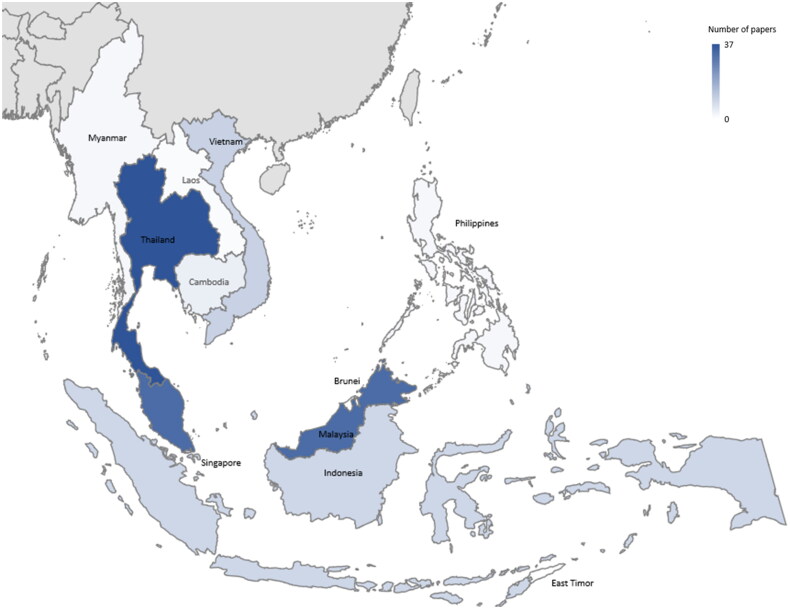
Geographical distribution of 108 selected publications on zoonotic pathogens in Southeast Asia.

Most of the 108 studies were conducted in Thailand (*n* = 37), followed by Malaysia (*n* = 32), Vietnam (*n* = 10), Indonesia (*n* = 9), Singapore (*n* = 5), Cambodia (*n* = 4), the Philippines (*n* = 2), Myanmar (*n* = 2) and Lao PDR (*n* = 1) (Figure 2). Several studies were conducted across multiple countries (*n* = 6). No studies were found from Brunei and Timor-Leste. We also noticed a substantial increase in publications in 2016 and after 2020, which appear to follow periods of outbreaks of major zoonotic diseases. However, the increase in number of publications may simply reflect the general increase in publications over that period.

Among 108 reviewed publications, most studies were focused on mammals (*n* = 89), followed by birds (*n* = 6), reptiles (*n* = 6), ticks collected from wild animals (*n* = 5), amphibians (*n* = 1), and mosquitoes collected near wild animal cages (*n* = 1). A total of 120 distinct pathogens were identified from 108 reviewed publications. Viruses were the most studied pathogens (*n* = 47), followed by parasites (*n* = 38) and bacteria (*n* = 35).

**Figure 3.**
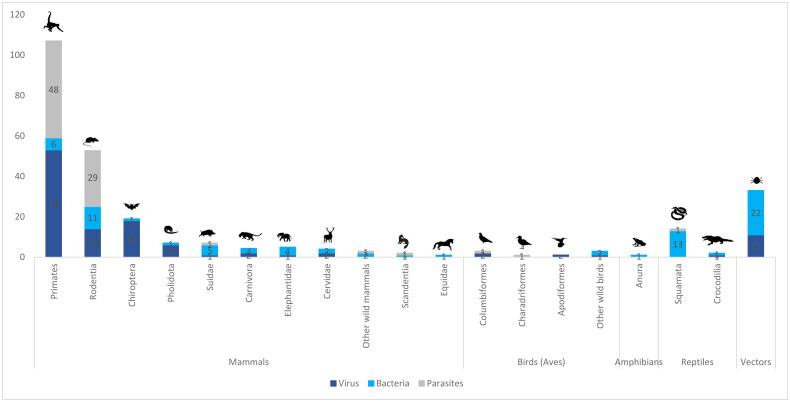
Number of studies on zoonotic pathogens and risk factors from wildlife in Southeast Asia (*n* = 273).

In our review comprising 108 publications, it is worth noting that a publication may 1) examine various pathogens within the same species; 2) investigate the same pathogen across different species; or 3) explore the same pathogens within the same species but from different sources (e.g. nature, captive); or 4) study the same pathogens but using different techniques (e.g. Polymerase Chain Reaction (PCR), Enzyme-linked immunosorbent assay (ELISA), culture). These are here referred to as different studies. We identified a total of 273 distinct studies within these 108 publications (Figure 3). In the following section (3.3), we organized the data according to individual studies. Each study represents a unique investigation, and by counting them individually, we can better capture the breadth and depth of the research conducted.

### 3.3. Zoonotic diseases associated with wildlife in Southeast Asia

Because animal hosts have different life-history traits or cycles that influence the potential spread of diseases in humans, it is important to understand the host-pathogen interface. The majority of studies examined pathogens from mammalian sources (80%, 214/273), others include reptilia (6%, 16/273), aves (3%, 9/273), amphibians (0.4%, 1/273). The tick studies covered 32 studies (12%, 32/273) while the mosquito study covered one study (0.4%, 1/273). Within mammals, primates received the most attention, accounting for 50% (107/214) of studies. This was followed by Rodentia (25%, 54/214), Chiroptera (9%, 20/214), Suidae (3%, 7/214), and Carnivora (2%, 5/214).

#### 3.3.1. Zoonotic diseases associated with primates in Southeast Asia

Because there were many studies (*n* = 107) related to primates in Southeast Asia, below we present results from studies with a positive rate exceeding the mean prevalence of all primate studies (which is equal to 22.5%) (Table 1), all primate studies can be found in Supplementary Table S2. The main pathogens studied were viruses (53 studies, 30 different viruses) of which the three most studied were herpesvirus, adenovirus, and chikungunya virus. Parasites were the next most frequently studied (48 studies, 23 different parasites) of which the three most studied were *Plasmodium* spp., *Trichuris* spp., and *Strongyloides* spp. Bacteria were studied the least (six studies, four different bacteria) which included *Bartonella* spp., *Mycobacterium* spp., *Leptospira* spp., and *Rickettsia* spp. Taken together, the five most commonly studied pathogens in primates were *Plasmodium* spp. *Trichuris* spp., *Strongyloides* spp., adenovirus, and herpesvirus. The five pathogens with the highest reported prevalence rates in primates over the past decade were *Plasmodium* spp. (96.1%), gastrointestinal parasites (89.6%), *Oesophagostomum* spp. (67.3%), measles virus (58.9%), and monkeypox virus (58.9%).

**Table 1.** List of studies focusing on zoonotic disease in primates in Southeast Asia.*

Pathogen	Country	Year of sampling	Sample size	% positive (95%CI)	Diagnostic test	Authors and published year
***Viruses***						
Adenovirus	Thailand	2013–2019	210	33.3 (27.1 − 40.2)	PCR	Kosoltanapiwat et al. (2022)
Anellovirus (captive primates)	Thailand	2016	43	25.6 (14 − 41.5)	PCR	Sawaswong et al. (2019)
Chikungunya virus	Vietnam	2014	90	53.3 (42.6 − 63.8)	ELISA	Zhang et al. (2016)
Dengue virus	Thailand	2018–2019	62	30.6 (19.9 − 43.8)	PRNT90	Tongthainan et al. (2020)
Epstein-Barr virus	Vietnam	2014	90	45.6 (35.1 − 56.4)	ELISA	Zhang et al. (2016)
Herpesvirus	Vietnam	2014	90	50 (39.9 − 60.1)	ELISA	Zhang et al. (2016)
Herpesvirus	Malaysia	2009–2011	149	49 (44.8 − 52.3)	ELISA	(Lee et al. 2015)
Herpesvirus	Malaysia	2009–2011	392	39.3 (34.5 − 44.3)	PCR	(Lee et al. 2015)
Japanese encephalitis virus	Indonesia	2019	92	43.4 (33.3 − 54.2)	ELISA	(Putra et al. 2022)
Measles virus	Vietnam	2014	90	58.9 (48 - 59)	ELISA	Zhang et al. (2016)
Monkeypox virus	Vietnam	2014	90	58.9 (48 - 59)	ELISA	Zhang et al. (2016)
Parvovirus (wild primates)	Thailand	2016	35	40 (23.4 − 57.8)	PCR	Sawaswong et al. (2019)
Rhesus cytomegalovirus	Vietnam	2014	90	58.9 (48 - 59)	ELISA	Zhang et al. (2016)
Rotavirus	Vietnam	2014	90	47.8 (37.2 − 58.5)	ELISA	Zhang et al. (2016)
Simian foamy virus	Vietnam	2014	90	56.7 (45.8 − 66.9)	ELISA	Zhang et al. (2016)
Simian foamy virus	Thailand	2020	649	56.5 (52.6 − 60.4)	PCR	Kaewchot et al. (2022)
Simian foamy virus	Cambodia	2006–2007	118	44.9 (35.8 − 54.3)	ELISA	Ayouba et al. (2013)
Simian T-lymphotropic virus	Vietnam	2014	90	53.3 (42.6 − 63.8)	ELISA	Zhang et al. (2016)
Simian type-D retrovirus	Vietnam	2014	90	53.3 (42.6 − 63.8)	ELISA	Zhang et al. (2016)
Simian varicella virus	Vietnam	2014	90	58.9 (48 - 59)	ELISA	Zhang et al. (2016)
West Nile Virus	Malaysia	2014–2017	81	28.6 (19.5 − 39.6)	ELISA	Ain-Najwa et al. (2020)
***Bacteria***						
*Leptospira* spp.	Thailand	2019	372	45.5 (4.3 − 50.6)	ELISA	Suwannarong et al. (2022)
*Mycobacterium avium*	Malaysia	2019–2020	30	33 (19.2 − 51.2)	PCR	Lekko et al. (2021)
***Parasites***						
*Entamoeba histolytica*	Singapore	2017	158	31.6 (24.6 − 39.6)	PCR	Heng et al. (2021)
Gastrointestinal parasites (Helminths and protozoans)	Malaysia	2012–2013	308	89.6 (85.5 − 92.7)	Microscopic examination	Adrus et al. (2019)
*Oesophagostomum* spp.	Indonesia	2004–2011	55	67.3 (53.2 − 78.9)	Microscopic examination	Yalcindag et al. (2021)
*Oesophagostomum* spp.	Indonesia	2004–2011	55	49.1 (35.5 − 62.8)	PCR	Yalcindag et al. (2021)
*Pedicinus* sp.	Malaysia	2016–2017	30	30 (154 − 49.6)	Microscopic examination	Choong et al. (2019)
*Plasmodium* spp.	Malaysia	2019–2021	152	96.1 (91.2 − 98.4)	PCR	Latif et al. (2022)
*Plasmodium* spp.	Malaysia	2016	103	62.1 (52 − 71.4)	PCR	Amir et al. (2020)
*Plasmodium* spp.	Malaysia	2014	70	50 (38.6 − 61.4)	PCR	Akter et al. (2015)
*Plasmodium* spp.	Philippines	2017	95	47.4 (37.1 − 57.8)	PCR	Gamalo et al. (2019)
*Plasmodium* spp.	Malaysia	2007–2010	46	36.9 (23.6 − 52.5)	PCR	Brown et al. (2022)
*Plasmodium* spp.	Singapore	2009–2017	1039	29.4 (26.6 − 32.2)	PCR	M.I. Li et al. (2021)
*Plasmodium* spp.	Thailand	2017–2019	93	29 (20.3 − 39.5)	PCR	Fungfuang et al. (2020)
*Plasmodium* spp.	Malaysia	2016	103	26.2 (18.3 - 36)	Microscopic examination	Amir et al. (2020)
*Strongyloides* sp.	Malaysia	2012	652	54.4 (50.5 − 58.3)	Microscopic examination	Klaus et al. (2017)
*Strongyloides* sp.	Thailand	2018	45	51.1 (36 − 66.1)	PCR	Janwan et al. (2020)
*Strongyloides* sp.	Thailand	2015	45	40 (26.1 − 55.6)	Faecal flotation and sedimentation	Teo et al. (2019)
*Strongyloides* sp.	Malaysia	2012	652	30 (26.6 − 33.8)	Microscopic examination	Klaus et al. (2017)
*Trichostrongylus* sp.	Malaysia	2016–2017	21	24 (9.1 − 47.5)	Faecal flotation and sedimentation	Choong et al. (2019)
*Trichuris* sp.	Malaysia	2012	652	79.8 (76.4 − 82.7)	Microscopic examination	Klaus et al. (2017)
*Trichuris* sp.	Thailand	2014–2017	315	48.5 (42.9 − 54.3)	PCR	Frias et al. (2021)
*Trichuris* sp.	Malaysia	2016–2017	21	38 (18.9 − 61.3)	Faecal flotation and sedimentation	Choong et al. (2019)

* Presentation of the 44 studies on primates that had prevalences above the mean value (22.5%).

CI: confidence interval; N/A: not available; PCR: Polymerase Chain Reaction; ELISA: Enzyme-linked immunosorbent assay; FECT: formalin-ether concentration technique; HI: hemagglutination-inhibition; PRNT90: 90% plaque reduction neutralization assay.

Of note, among the reviewed publications, nine searched for 14 pathogens, including viruses (chikungunya virus, dengue virus, Ebola virus, hepatitis A virus, hepatitis B virus, hepatitis C virus, picornaviruses, retrovirus, West Nile Virus, Zika virus), bacteria (*Mycobacterium* spp. and *Rickettsia* spp.), and parasites (helminth communities and *Cryptosporidium* spp.) but could not find evidence of their presence.

The majority of these studies were conducted in Thailand (*n* = 45), followed by Malaysia (*n* = 27), Vietnam (*n* = 21), and Indonesia (*n* = 9). Other Southeast Asian countries also contributed data, with Singapore reporting two studies, and Cambodia and the Philippines reporting one study each.

#### 3.3.2. Zoonotic diseases associated with rodentia in Southeast Asia

There were many studies (*n* = 54) on zoonotic diseases in *Rodentia.* Similar to above, we only present the studies with a positive rate % exceeding the mean prevalence of all studies of *Rodentia* (which is equal to 24.8%) (Table 2), with all studies can be found in Supplementary Table S3. The main pathogen studied was parasites (29 studies, 20 different parasites) of which the three most studied were helminth communities, *Trypanosoma lewisi,* and *Giardia* spp. Viruses were the next most frequently studied (14 studies, nine different viruses) of which the three most studied were hepatitis E, hantavirus, and coronavirus. Bacteria were studied the least (11 studies, five different bacteria) including seven studies on *Leptospira* spp., and one study for each following bacteria *Borrelia* spp., *Orientia tsutsugamushi*, *Rickettsia* spp., *Salmonella* spp. Taken together, the five most commonly studied pathogens in rodents were hepatitis E virus, hantavirus, *Leptospira* spp., helminth communities, and *Trypanosoma lewisi*. The five pathogens with the highest prevalence rates in *Rodentia* over the past decade were *Strongyloides* spp. (81.3%), helminth communities (80.0%), *Rickettsia* spp. (78.0%), ectoparasites (72.7%), and *Nippostrongylus brasiliensis* (35.0%).

**Table 2.** List of studies focusing on zoonotic disease in Rodentia in Southeast Asia.*

Pathogen	Country	Year of sampling	Sample size	% positive (95%CI)	Diagnostic test	Authors and published year
***Viruses***						
Coronavirus	Vietnam	2013-2014	702	34.04 (30.6 − 37.7)	PCR	Huong et al. (2020)
Hepatitis E virus	Indonesia	2012	369	37.1 (32.2 − 42.3)	ELISA	Mulyanto et al. (2014)
Hepatitis E virus	Indonesia	2012	369	26.3 (21.9 − 31.1)	PCR	Mulyanto et al. (2014)
***Bacteria***						
*Leptospira* spp.	Philippines	2013-2014	51	39.2 (26.2 − 53.9)	Culture	N. Gloriani et al. (2016)
*Leptospira* spp.	Cambodia, Vietnam	2019	72	38.9 (27.8 − 51.1)	PCR	Koizumi et al. (2022)
*Leptospira* spp.	Indonesia	2019	80	31.3 (21.6 − 42.7)	MAT and sequencing	Koizumi et al. (2022)
*Rickettsia* spp.	Thailand	2015-2016	237	78 (72.1 - 83)	ELISA	Prompiram et al. (2020)
*Salmonella* spp.	Thailand	2014	54	49.1 (39.4 − 58.8)	PCR	Ribas et al. (2016)
***Parasites***						
Ectoparasites	Malaysia	2015-2016	22	72.7 (49.7 − 88.4)	Microscopic examination	Ahmad et al. (2020)
*Giardia* spp.	Malaysia	2019	32	28.1 (14.4 - 47)	Fecal flotation	Mohd-Qawiem et al. (2022)
Helminth communities	Malaysia	2000-2002	485	80 (76.1 − 83.4)	Microscope examination	Zain et al. (2012)
Helminth communities	Lao PDR	N/A	404	65.8 (61 − 70.4)	Microscopic examination	Pakdeenarong et al. (2014)
Helminth communities	Cambodia	2008-2014	569	58.5 (54.3 − 62.6)	Microscopic examination	Chaisiri et al. (2017)
Helminth communities	Thailand	2008-2012	2478	29.7 (27.9 − 31.5)	Microscopic examination	Chaisiri et al. (2015)
*Hymenolepis nana*	Malaysia	2019	32	46.9 (29.5 - 65)	Fecal flotation	Mohd-Qawiem et al. (2022)
*Nippostrongylus brasiliensis*	Malaysia	2019	32	53.1 (35 − 70.5)	Fecal flotation	Mohd-Qawiem et al. (2022)
*Ornithonyssus* spp.	Malaysia	2019	32	53.1 (35 − 70.5)	Microscopic examination	Mohd-Qawiem et al. (2022)
*Pneumocystis* spp.	Thailand, Cambodia, Lao PDR, Philippines	2011-2015	731	43.6 (40 − 47.3)	PCR	Latinne et al. (2021)
*Strongyloides* sp.	Malaysia	2019	32	81.3 (63 − 92.1)	Fecal flotation	Mohd-Qawiem et al. (2022)
*Xenopsylla cheopis*	Malaysia	2019	32	43.8 (26.8 − 62.1)	Microscopic examination	Mohd-Qawiem et al. (2022)

* Presentation of the 20 studies on Rodentia that had prevalences above the mean value (24.8%).

CI: confidence interval; N/A: not available; PCR: Polymerase Chain Reaction; ELISA: Enzyme-linked immunosorbent assay; MAT: Modified agglutination test.

Notably, two studies searched for *Yersinia pestis* and rodent parechoviruses (Ljungan virus) in Rodentia in Vietnam, but could not find any evidence of their presence.

The majority of studies were conducted in Malaysia (*n* = 24), followed by Vietnam (*n* = 14), and Thailand (*n* = 7). Other Southeast Asian countries also contributed data, with Indonesia reporting three studies, Cambodia reporting two studies, and the Philippines, Lao People’s Democratic Republic (PDR), and Singapore reporting one study each. Two studies involved multiple countries researched two pathogens *Leptospira* spp. and *Pneumocystis* spp.

#### 3.3.3. Zoonotic diseases associated with chiroptera in Southeast Asia

Our research revealed 20 studies on potential zoonotic diseases linked to bats (Chiroptera) (Table 3). These include 18 studies on seven different viruses (10 studies on coronaviruses, two studies on Nipah virus, two studies on astrovirus, and one study for each following virus: Ebola virus, picornaviruses, West Nile virus, polyomaviruses; one study on one bacteria (*Bartonella* spp.) and one study on ectoparasites. Taken together, the three most commonly studied pathogens in *Chiroptera* were coronavirus, Nipah virus, and astrovirus. The five pathogens with the highest prevalence rates in *Chiroptera* over the past decade were coronavirus (74.8%), picornavirus (67.0%), ectoparasites (65.7%), astroviruses (55%), and *Bartonella* spp. (25.1%).

**Table 3.** List of studies focusing on zoonotic disease in *Chiroptera* in Southeast Asia.

Pathogen	Country	Year of sampling	Sample size	% positive (95%CI)	Diagnostic test	Authors and published year
***Viruses***						
Astroviruses	Singapore	2011-2015	169	55 (47.2 − 62.6)	PCR	Mendenhall et al. (2017)
Astroviruses	Lao PDR	2010-2013	1997	4.7 (3.8 − 5.7)	PCR	Afelt et al. (2018)
Coronavirus	Vietnam	2013-2014	313	74.8 (69.5 − 79.4)	PCR	Huong et al. (2020)
Coronavirus	Thailand	2012	367	18.5 (14.8 - 23)	PCR	(Wacharapluesadee et al. (2018)
Coronavirus	Thailand	2020	100	13 (7.4 − 21.6)	PCR	(Wacharapluesadee et al. (2021)
Coronavirus	Thailand	2008-2013	629	7.5 (5.6 − 9.9)	PCR	Wacharapluesadee et al. (2015)
Coronavirus	Myanmar	2016-2018	759	6.3 (4.7 − 8.4)	PCR	Valitutto et al. (2020)
Coronavirus	Cambodia	2010-2013	1997	4.7 (3.8 − 5.7)	PCR	Afelt et al. (2018)
Coronavirus	Lao PDR, Cambodia	2010-2013	1965	4.7 (3.9 − 5.8)	PCR	Lacroix et al. (2017)
Coronavirus	Thailand	2020	98	4.1 (1.3 − 10.7)	ELISA	Wacharapluesadee et al. (2021)
Coronavirus	Cambodia	2010	10	0.4 (13.7 − 72.6)	PCR	Zhu et al. (2022)
Coronavirus	Myanmar	2018	29	0 (0 − 14.6)	PCR	McEvoy et al. (2021)
Ebola virus	Thailand	2011-2013	699	0 (0 − 0.7)	ELISA and PCR	(Wacharapluesadee et al. (2015)
Nipah virus	Thailand	2002-2020	2500	5.6 (4.7 − 6.5)	PCR	(Wacharapluesadee et al. (2021)
Nipah virus	Thailand	2010-2011	184	2.7 (1 − 6.6)	PCR	Wacharapluesadee et al. (2016)
Picornaviruses	Vietnam	2012-2016	179	67 (59.6 − 73.8)	PCR	Lu et al. (2021)
Polyomaviruses	Indonesia	2012-2013	88	11.4 (5.9 − 20.3)	PCR	Kobayashi et al. (2015)
West Nile Virus	Malaysia	2014-2017	41	12.2 (4.6 - 27)	PCR	Ain-Najwa et al. (2020)
***Bacteria***						
*Bartonella* spp.	Thailand	2018-2020	459	25.1 (21.2 − 29.3)	PCR	Poofery et al. (2022)
***Parasites***						
Ectoparasites	Malaysia	2015-2016	35	65.7 (47.7 − 80.3)	Microscopic examination	Ahmad et al. (2020)

CI: confidence interval; PCR: Polymerase Chain Reaction; ELISA: Enzyme-linked immunosorbent assay.

Two studies searched for coronaviruses in bats in Myanmar and Ebola virus in bats in Thailand but could not find any evidence of their presence.

The majority of studies were conducted in Thailand (*n* = 8), followed by Vietnam (*n* = 2), while the remaining studies are distributed across the region. Three studies have been conducted in multiple countries (two studies on coronavirus and one study on astrovirus).

#### 3.3.4. Zoonotic diseases associated with Suidae in Southeast Asia

Among seven studies related to *Suidae* in Southeast Asia (Table 4), the main pathogen studied were bacteria *(*three studies on *Mycobacterium* spp., one study on *Leptospira* spp. and one study on *Anaplasma* spp.); one study investigated *Trichinella* species, a type of parasite transmissible from wild boars to humans. The three pathogens with the highest prevalence rates in *Suidae* over the past decade were *Mycobacterium avium* (91.0%), *Mycobacterium tuberculosis* (75.0%), and *Anaplasma* spp. (70.0%).

**Table 4.** List of studies focusing on zoonotic disease in *Suidae* in Southeast Asia.

Pathogen	Country	Year of sampling	Sample size	% positive (95%CI)	Diagnostic test	Authors and published year
***Viruses***						
Picornaviruses	Vietnam	2012-2016	15	0 (0 − 25.3)	PCR	Lu et al. (2021)
***Bacteria***						
*Anaplasma* spp.	Malaysia	2013-2015	10	70 (35.4 − 91.9)	PCR	Koh et al. (2016)
*Leptospira* spp.	Thailand	2009	58	62.1 (48.3 − 74.2)	MAT	Prompiram et al. (2019)
*Mycobacterium avium*	Malaysia	2019-2020	12	91 (64.6 − 98.5)	PCR	Lekko et al. (2021)
*Mycobacterium tuberculosis*	Malaysia	2019-2020	12	75 (46.8 − 91.1)	PCR	Lekko et al. (2021)
*Mycobacterium tuberculosis*	Malaysia	2019-2020	30	16.7 (7.3 − 33.5)	ELISA	Lekko et al. (2021)
***Parasites***						
*Trichinella* spp.	Vietnam	2010-2013	62	3.2 (0.8 − 4.8)	PCR	Thi et al. (2014)

CI: confidence interval; PCR: Polymerase Chain Reaction; ELISA: Enzyme-linked immunosorbent assay; MAT: Modified agglutination test.

The majority of studies were conducted in Malaysia (*n* = 4). Other Southeast Asian countries also contributed data were Vietnam (*n* = 2) and Thailand (*n* = 1). Another study conducted in Vietnam screened for a virus (Picornavirus) but could not detect this in wild boars.

#### 3.3.5. Zoonotic diseases associated with carnivora in Southeast Asia

Research on zoonotic diseases from carnivores in Southeast Asia was limited, with only five studies identified in the past decade (Table 5). The main pathogens studied were bacteria (*Rickettsia* spp. and *Bartonella* spp.) and viruses (avian influenza virus and SARS-related coronaviruses). The two pathogens with the highest prevalence rates in *Carnivora* over the past decade were avian influenza virus (10.2%) and *Bartonella* spp. (6.7%).

**Table 5.** List of studies focusing on zoonotic disease in *Carnivora* in Southeast Asia.

Pathogen	Country	Species	Year of sampling	Sample size	% positive (95%CI)	Diagnostic test	Authors and published year
***Viruses***							
Avian Influenza virus	Thailand	Captive felid	2011-2015	196	10.2 (6.5 − 15.5)	ELISA	Sangkachai et al. (2019)
SARS-related coronaviruses	Vietnam	Civets	2017-2019	299	0 (0 - 0)	PCR	Nga et al. (2022)
***Bacteria***							
*Bartonella* spp.	Malaysia	Foxes	2016	30	6.67 (1.2 − 23.5)	PCR	Hou et al. (2018)
*Rickettsia* spp.	Malaysia	Sun bears	2019	6	0 (0 - 0)	PCR	Low et al. (2022)
*Rickettsia* spp.	Malaysia	Tigers	2019	6	0 (0 - 0)	PCR	Low et al. (2022)

CI: confidence interval; PCR: Polymerase Chain Reaction; ELISA: Enzyme-linked immunosorbent assay.

Among those, three studies on two pathogens, including SARS-related coronaviruses in civets and *Rickettsia* spp. in tigers and bears, did not find evidence of these pathogens in these animals.

The majority of studies were conducted in Malaysia (*n* = 3). Other Southeast Asian countries also contributed data were Vietnam (*n* = 1) and Thailand (*n* = 1).

#### 3.3.6. Zoonotic diseases associated with vectors collected from or near wild animals in Southeast Asia

This review further investigated the role of vectors in zoonotic diseases in Southeast Asia. "Vectors collected from or near wild animals" refers to organisms capable of transmitting pathogens that are sampled directly from wild animals (e.g. ticks attached to a host) or from their surrounding environment (e.g. mosquitoes near wildlife habitats). Among 33 studies related to vectors collected from or near wild animals (Table 6), the main pathogens studied were bacteria from ticks removed from wild animals (wild boars and rodents) (22 studies, 17 different bacteria) of which the three most studied were *Coxiella* (including *Coxiella burnetii* and *Coxiella-*like bacteria), *Rickettsia* spp., and *Staphylococcus* spp. Viruses from ticks collected from wild animals (wild boars and rodents) were next most frequently studied (11 studies, six different viruses) of which the three most studied were chuviruses, flaviviruses, and orthomyxoviruses. Taken together, the five most commonly studied pathogens associated with vectors collected from or near wild animals were *Coxiella*, chuviruses, flaviviruses, orthomyxoviruses, and phenuiviruses. The five most prevalent pathogens identified over the past decade in vectors collected from or near wild animals were *Coxiella* in ticks collected from porcupine carcasses (100%), *Coxiella* in ticks (*Haemaphysalis hystricis*) collected from wild boar carcasses (100%), *Coxiella* in ticks (*Dermacentor compactus*) collected from wild boar carcasses (100%), *Borrelia* spp. in ticks collected from rodents (50%) and *Coxiella* in ticks (*Dermacentor steini*) collected from wild boar carcasses (100%).

**Table 6.** List of studies focusing on zoonotic diseases associated with vectors collected from or near wild animals in Southeast Asia.

Pathogen	Country	Vectors (ticks or mosquitoes) from	Year of sampling	Sample size	% positive (95%CI)	Diagnostic test	Authors and published year
***Viruses***							
Arbovirus	Indonesia	Mosquitoes collection around long-tailed macaques cages	2021	1108	0 (0 − 0.4)	PCR	Novianto et al. (2022)
Chuviruses	Thailand	Ticks removed from wild boars *(all vectors below are ticks collected from)*	2012	62	0 (0 − 7.3)	PCR	Temmam et al. (2019)
Chuviruses	Thailand	Rodents	2012	1	0 (0 − 94.5)	PCR	Temmam et al. (2019)
Flaviviruses	Thailand	Wild boars	2012	62	0 (0 − 7.3)	PCR	Temmam et al. (2019)
Flaviviruses	Thailand	Rodents	2012	1	0 (0 − 94.5)	PCR	Temmam et al. (2019)
Orthomyxoviruses	Thailand	Wild boars	2012	62	0 (0 − 7.3)	PCR	Temmam et al. (2019)
Orthomyxoviruses	Thailand	Rodents	2012	1	0 (0 − 94.5)	PCR	Temmam et al. (2019)
Phenuiviruses	Thailand	Wild boars	2012	62	0 (0 − 7.3)	PCR	Temmam et al. (2019)
Phenuiviruses	Thailand	Rodents	2012	1	0 (0 − 94.5)	PCR	Temmam et al. (2019)
Rhabdoviruses	Thailand	Wild boars	2012	62	0 (0 − 7.3)	PCR	Temmam et al. (2019)
Rhabdoviruses	Thailand	Rodents	2012	1	0 (0 − 94.5)	PCR	Temmam et al. (2019)
***Bacteria***							
*Acinetobacter*	Malaysia	Wild boar carcasses	2014	72	4.4 (1.1 − 12.5)	PCR	Lim et al. (2020)
*Actinomycetia*	Malaysia	Wild boar carcasses	2014	72	1.7 (0.1 − 8.5)	PCR	Lim et al. (2020)
*Arthrobacter*	Malaysia	Wild boar carcasses	2014	72	1.7 (0.1 − 8.5)	PCR	Lim et al. (2020)
*Bacillales*	Malaysia	Wild boar carcasses	2014	72	4.2 (1.1 − 12.5)	PCR	Lim et al. (2020)
*Borrelia* spp.	Malaysia	Rodents	2018-2019	32	43.7 (26.8 − 62.1)	PCR	Lau et al. (2020)
*Brevibacterium*	Malaysia	Wild boar carcasses	2014	72	1.2 (0.1 − 8.5)	PCR	Lim et al. (2020)
*Corynebacterium*	Malaysia	Wild boar carcasses	2014	72	2.1 (0.1 − 10.6)	PCR	Lim et al. (2020)
*Coxiella*	Malaysia	Porcupine carcasses	2014-2015	6	100 (51.7 - 100)	PCR	Khoo et al. (2016)
*Coxiella*	Malaysia	Wild boar carcasses (Haemaphysalis hystricis)	2014-2015	19	100 (51.7 - 100)	PCR	Khoo et al. (2016)
*Coxiella*	Malaysia	Wild boar carcasses (Dermacentor compactus)	2014-2015	2	50 (9.4 − 90.5)	PCR	Khoo et al. (2016)
*Coxiella*	Malaysia	Wild boar carcasses (Dermacentor steini)	2014-2015	7	28.6 (5.1 − 69.7)	PCR	Khoo et al. (2016)
*Coxiella*	Malaysia	Wild boar carcasses	2014	72	16.6 (9.3 − 27.7)	PCR	Lim et al. (2020)
*Enterobacteriaceae*	Malaysia	Wild boar carcasses	2014	72	2.9 (0.1 − 10.6)	PCR	Lim et al. (2020)
*Erwinia*	Malaysia	Wild boar carcasses	2014	72	4.1 (1.1 − 12.5)	PCR	Lim et al. (2020)
*Francisella*	Malaysia	Wild boar carcasses	2014	72	5.4 (1.8 − 14.3)	PCR	Lim et al. (2020)
*Gammaproteobacteria*	Malaysia	Wild boar carcasses	2014	72	3.6 (1.1 − 12.5)	PCR	Lim et al. (2020)
*Pseudomonas* spp.	Malaysia	Wild boar carcasses	2014	72	1.7 (0.1 − 8.5)	PCR	Lim et al. (2020)
*Rickettsia* spp.	Malaysia	Wild boar carcasses	2014	72	9.1 (4.3 − 19.6)	PCR	Lim et al. (2020)
*Rickettsia* spp.	Thailand	Burmese ferret-badger (Haemaphysalis hystricis)	2019	16	6.3 (0.3 − 32.3)	PCR	Hirunkanokpun et al. (2022)
*Staphylococcaceae*	Malaysia	Wild boar carcasses	2014	72	10.9 (5.3 − 21.3)	PCR	Lim et al. (2020)
*Staphylococcus* spp.	Malaysia	Wild boar carcasses	2014	72	14.7 (8.2 − 26.1)	PCR	Lim et al. (2020)
*Stenotrophomonas*	Malaysia	Wild boar carcasses	2014	72	2.1 (0.1 − 10.6)	PCR	Lim et al. (2020)

CI: confidence interval; PCR: Polymerase Chain Reaction.

Notably, 11 studies found no evidence of six zoonotic viral pathogens (chuviruses, flaviviruses, orthomyxoviruses, phenuiviruses, rhabdoviruses) in ticks from wild boars and rodents, nor arboviruses in mosquitoes from wild animal habitats.

The majority of studies on zoonotic diseases associated with vectors were conducted in Malaysia (*n* = 21), followed by Thailand (*n* = 11), and Indonesia (*n* = 1).

### 3.4. Key risk factors for zoonotic disease transmission in Southeast Asia

From the 108 publications, we identified 11 risk factors influencing zoonotic pathogen transmission. This synthesis does not involve new statistical analysis but rather reflects patterns reported in the literature. We further investigated publications that simultaneously studied human exposure alongside animal models. A total of 30 out of 108 publications reviewed (28%) included this simultaneous evaluation. Table 7 presents an overview of the risk factors which we categorized under four broad categories (environmental factors, animal factors, human factors, human-animal-environmental interface) while Supplementary Table S4 presents a detailed list of each zoonotic pathogen and their risk factors and wildlife host.

**Table 7.** Risk factors for zoonotic pathogen transmission from wildlife identified in Southeast Asia.

Categories	Risk factors	Type of wildlife animals
Environmental factors	Animal habitat disruption	Primates, *Rodentia, Chiroptera*
	Environment conditions	Primates, *Rodentia,* ticks in rodents and wild boars*, Carnivora*
	Exposure to contaminated water/food/soil	Primates, *Rodentia, Chiroptera, Suidae* and other wild mammals
Animal factors	Animal movement patterns	*Chiroptera* and wild birds
	Animal age	Primates, *Chiroptera*
Human factors	Lack of awareness and knowledge, poor hygiene practices	Primates, *Rodentia, Chiroptera, Suidae, Anura*
	Gender and income	Primates
	Human age	*Elephantidae*
Human-animal-environmental interface factors	Close contact between humans and animals	Primates, *Rodentia, Chiroptera, Carnivora, Suidae, Pholidota, Squamata* and wild birds
	Exposure through humans deliberately visiting animals	Primates, *Carnivora*
	Presence of vectors in human habitat	Primates, *Rodentia*

#### 3.4.1. Environmental factors

The majority of the reviewed publications reported heightened risks associated with disruptions to the natural habitats of wild animals, often caused by human encroachment (Tongthainan et al. 2020; Valitutto et al. 2020; McEvoy et al. 2021). Others presented factors such as deforestation, urbanization, hunting, and environmental disturbances (Afelt et al. 2018; Ain-Najwa et al. 2020; Amir et al. 2020; Tongthainan et al. 2020; McEvoy et al. 2021; Yalcindag et al. 2021). These factors may increase opportunities for contact between different wildlife species, livestock, and humans, consequently increasing disease transmission among them.

Some publications emphasized environmental conditions as a key risk factor for the transmission of zoonotic pathogens to humans. For example, Temmam et al. (2019) found that global and local environmental changes and the geographical repartition of ticks are increasing, leading to the exposure of naïve populations to new pathogens. Prompiram et al. (2022), Low et al. (2022) and Lau et al. (2020) identified that warm and humid weather represents a period of greater zoonotic risk. According to these authors, the tropical climate (warm and humid weather) created a favorable environment for infected ticks and fleas that increase the exposure of humans to these vectors and their reservoirs, therefore, posing a zoonotic threat to the local population, especially animal handlers, and farm workers.

Some publications highlighted that humans or animals can be infected through contaminated environmental media such as water, food, and/or soil (Latifah et al. 2012; Koizumi et al. 2015; N.G. Gloriani et al. 2016; Wacharapluesadee et al. 2018; Kudo et al. 2018; Janwan et al. 2020; Heng et al. 2021; Lekko et al. 2021).

We found that animal habitat disruption was the main risk factor covered in the primate*, Rodentia, Chiroptera* studies. Environmental conditions were the key risk factor identified in the primates, *Rodendia, Carnivora,* and ticks collected in rodents and wild boars studies, while exposure to contaminated environments (water, food, or soil) was a concern across studies investigating zoonotic diseases in primates, *Rodentia*, *Chiroptera, Suidae,* and other small wild mammals.

#### 3.4.2. Animal factors

Research has shown a link between the movement of wild birds and the spread of viruses like highly pathogenic avian influenza H5N1 to domestic animals and humans. Studies by Takakuwa et al. (2013) and Mohamed et al. (2022) highlighted this connection. As migratory birds come into contact with poultry farms and human settlements, they can act as potential carriers and transmitters of these viruses. The age of animals also may play a role as a potential risk factor, Mendenhall et al. (2017) observed that younger bats exhibited a lower prevalence of astroviruses, suggesting they may be less susceptible to infection. Another study conducted by Wacharapluesadee et al. (2018) found that CoV infection was found to be associated with bats of younger age. Kaewchot et al. (2022) reported an increase in simian foamy virus prevalence in male non-human primates, compared to females.

Findings from our review suggest that animal movement patterns were a major factor for zoonotic disease transmission in flying animals, particularly *Chiroptera* (bats) and wild birds. Additionally, age appeared to be a risk factor in both *Chiroptera* and primates.

#### 3.4.3. Human factors

Our review suggested that human sociodemographic factors such as gender, income, and age can influence susceptibility to zoonotic diseases, but we could only find two studies for this review. One study reported that being older than 30 years old increased the risk of tuberculosis (Yakubu et al. 2016) and being male and having a low household income was positively correlated with the occurrence of *Leptospira* (Suwannarong et al. 2022). Additionally, human behavioral factors like consuming raw or undercooked meat from wild animals (Thi et al. 2014; Lacroix et al. 2017), handling infected animal fluids (Ayouba et al. 2013), consuming contaminated food and vegetables (Ribas et al. 2016; Wacharapluesadee et al. 2018; Mohd Fadil et al. 2019; Nguyen et al. 2021), and lacking awareness about local zoonotic diseases (Choong et al. 2019), all contributed to increased transmission risks. These studies highlighted the importance of promoting safe food handling practices and raising awareness about zoonotic disease risks in affected communities.

We found that lack of awareness and knowledge, along with poor hygiene practices, were the main risk factors identified in studies on primates, *Rodentia, Chiroptera*, *Suidae,* and *Anura*. Human age, gender, and income emerged as additional risk factors in studies on primates and elephants (*Elephantidae*).

#### 3.4.4. Human-animal-environmental interface factors

Close and frequent contact between wild animals and zookeepers, farmers, hunters, and traders can increase the risk of transmitting zoonotic diseases from animals to humans or vice versa. This risk factor has been reported in many of the reviewed publications. Increased risk of transmission was reported from various activities such as wildlife farming (Azhari et al. 2018; Huong et al. 2020), feeding (Smith et al. 2012; Teo et al. 2019), trading (Smith et al. 2012; Ribas et al. 2016; Huong et al. 2020; Nga et al. 2022), handling carcasses of dead animals (Lee et al. 2015, p. 2009–2011; Ribas et al. 2016; Abba et al. 2017), and consuming (Van Cuong et al. 2015; Ribas et al. 2016). Smith et al. (2012), Ribas et al. (2016), Huong et al. (2020), Lee et al. (2020), Nga et al. (2022) highlighted the risks of transmission of zoonotic pathogens during the wildlife trade transits when animals are often housed together in groups from disparate geographic regions, and often with other species, giving opportunity for viral transmission between and within species. Wildlife in markets have a much higher chance of both exposure to pathogens and disease spillover. Additionally, having people visit and spend time with wild animals at the zoo or public areas increases proximity and time of contact with animals, making these places important transmission zones for zoonotic diseases (Koompapong et al. 2014; Sangkachai et al. 2019; Chaiwattanarungruengpaisan et al. 2021; Kosoltanapiwat et al. 2022; Prompiram et al. 2022). Moreover, the presence of disease-carrying vectors in human habitats, like mosquitoes (Akter et al. 2015; Fungfuang et al. 2020; Novianto et al. 2022) or ticks (Mohd-Qawiem et al. 2022), was also identified as a risk factor for zoonotic transmission.

We found that close contact between humans and animals was the main risk factor covered in the studies of primates, *Rodentia, Chiroptera, Carnivora, Suidae, Pholidota, Squamata,* and wild birds. Exposure through humans deliberately visiting animals was another key risk factor identified in the primate and *Carnivora* studies while the presence of vectors in human habitat was a concern across studies investigating zoonotic diseases associated with vectors collected from or near primates and *Rodentia*.

## 4. Discussion

This systematic literature review examined publications on wildlife zoonoses in Southeast Asia. We found a strong focus on mammals, which is not surprising considering most emerging human diseases originate from mammalian hosts (Morse et al. 2012). Notably, none of the reviewed studies included animals from more than one class, unlike what has been found in other topics, such as disease emergence in relation to urbanization and land-use change (Hassell et al. 2017; White and Razgour 2020). As such, research identifying spillover risks across taxonomic boundaries is important for addressing future zoonotic threats.

The publications reviewed mainly reported on wildlife zoonoses in Thailand, Malaysia, Vietnam, and Indonesia. This trend could maybe be explained by the presence of major international institutions working in One Health, including the World Health Organization (WHO), Food and Agriculture Organization (FAO), World Organization for Animal Health (WOAH), and United Nations Environment Programme (UNEP) in these countries. Additionally, some of these countries have a history of taking action, exemplified by initiatives like the establishment of the Ministerial Committee on the Control of Zoonotic Diseases in Malaysia in 1999 (WHO 2020a). This review also highlights gaps in zoonotic research in countries like Brunei and Timor-Leste, potentially pointing to the lack of resources or research priority. Furthermore, there were limited studies reporting on zoonoses across countries, hindering our ability to understand the transmission risks regionally. Strengthening regional, multisectoral collaboration and knowledge sharing is needed, and the Association of Southeast Asian Nations (ASEAN)’s commitment to establishing the ASEAN One Health Network is an encouraging step that requires timely follow-through (ASEAN 2023).

The review highlights the presence of a wide variety of zoonotic pathogens across different wildlife species in Southeast Asia, indicating a multifaceted risk landscape. Importantly, the identification of several emerging and re-emerging pathogens, with reportedly high prevalence rates in recent years – such as *Plasmodium* spp. in primates (96.1%) (Latif et al. 2022), *Strongyloides* spp. in wild rats (81.3%) (Mohd-Qawiem et al. 2022), *Rickettsia* spp. in rodents (78.0%) (Prompiram et al. 2020), coronaviruses in bats (74.8%) (Huong et al. 2020) and *Leptospira* in wild boars (62.1%) (Prompiram et al. 2019) – underscores the ongoing threat posed by these diseases to both animal and human health in the region. The high prevalence rates also emphasize the importance of robust surveillance and monitoring to track zoonotic diseases in their animal reservoirs, identifying potential human hosts, and addressing the factors that contribute to spillover.

Coronaviruses have caused significant outbreaks and pandemics, such as the COVID-19 pandemic caused by SARS-CoV-2, which warrant attention. A study by Huong et al. (2020) in Vietnam found that at sites where human-wildlife interactions occur, such as bat farms and field rat trade, where a high percentage of coronaviruses were detected in bats (74.8%) and to a lesser extent rodents (34.0%). This close contact between humans and animals harboring diverse viral strains significantly increases the risk of zoonotic spillover events. Furthermore, the study suggested that the confined conditions can act as a breeding ground for viral amplification and potentially even recombination, leading to potentially even more dangerous virus strains. This risk is likely not limited to bats and rodents but also extends to other wildlife supply chains where diverse animals like civets and pangolins are transported and confined; however, these supply chains receive relatively little attention (Nga et al. 2022). Additionally, the detection of coronaviruses in farmed rodents and bats sheds light on risks for intra-species transmission associated with agri-food production systems that involve close contact with these animals (Huong et al. 2020). Overall, these findings suggest the need for targeted preventive measures within wildlife value chains.

The lack of research on hantaviruses in Southeast Asia is concerning, with only three studies conducted in Vietnam in the past decade. Unlike coronaviruses, which have sparked global pandemics, hantaviruses are typically associated with localized outbreaks, often in areas where humans come in contact with rodent populations. However, hantaviruses can lead to severe illness, including kidney failure and hemorrhage. Van Cuong et al. (2015) found a considerable hantavirus seroprevalence (8.3%) among rodent species in southern Vietnam, similarly a study conducted by Blasdell et al. (2011) in Cambodia, Lao PDR, and Thailand found up to 5.6% of rodents carrying hantavirus antibodies, highlighting the need for targeted surveillance and control measures in areas where rodents are commonly trapped and consumed.

Similarly, despite Southeast Asia having a high burden of hepatitis E compared to other regions globally (WHO 2023), research on hepatitis E remains limited, with only five studies conducted. Hepatitis E is a major cause of acute viral hepatitis. The review found this virus can infect humans through the consumption of contaminated water or raw or undercooked food products from wild animals such as wild boar (Li et al. 2005) or deer (Tei et al. 2003). Pig farmers and veterinarians show markedly higher hepatitis E seroprevalence compared to the general public, hinting at occupational exposure risk (Krumbholz et al. 2012). A reported case of rat hepatitis E (Orthohepevirus-C) in a human raises concern about rodent reservoirs and zoonotic transmission (Sridhar et al. 2021). Further studying this virus in wild animals is important for understanding the transmission dynamics, and subsequently, preventative measures to address this issue.

This systematic literature review provided a comprehensive analysis of zoonotic disease transmission in Southeast Asia, highlighting key risk factors and their implications for public health and wildlife management. By exploring the complex interplay between environmental, animal, human, and human-animal-environmental interface factors, our findings offered valuable insights for developing targeted interventions to mitigate zoonotic risks in the region.Over the past three decades, Southeast Asia has seen a surge in both domestic and international demand for wild meat (Taylor et al. 2001; Cutler et al. 2010). To meet this growing demand, commercial wildlife farming has proliferated rapidly in terms of farm numbers, species diversity, and scale of operations. A 2014 survey in southern Vietnam exemplifies this trend, revealing nearly 1 million wild animals being raised across 4,099 active farms (FAO 2014). These farms often house a diverse mix of species, including rodents, primates, wild boars, and even reptiles, and frequently co-raise them with domestic animals. This close proximity between wild and domestic animals on farms, combined with the hunting and trade of wild animals in wet markets and restaurants (Volpato et al. 2020) raises significant concerns for zoonotic disease transmission. As highlighted by Recht et al. (2020), human interactions with wild animals throughout the supply chain, from hunting and farming to preparation and consumption of uncooked meat, can facilitate the emergence and spread of important zoonotic diseases. However, despite this evident risk, research on the prevalence and transmission of zoonotic diseases within Southeast Asian wildlife farms, as well as the transmission dynamics between wild and domestic animals and humans, remains scarce. To effectively manage these risks, urgent research is needed to determine the specific zoonotic threats associated with key wildlife species throughout the entire wildlife supply chain.

The data from reviewed studies suggested that wildlife continues to play a significant role in zoonotic disease transmission in Southeast Asia, however, when investigating risk factors it is important to consider both human and animal studies together. Among the 108 publications examined, only 30 included data also on human exposure. This limited focus on human data represents a significant gap in the current research. Moreover, given the potential variability in transmission routes and risk factors across different countries, wildlife species, and pathogens, employing more sensitive and comprehensive research methodologies to investigate exposure in both humans and animals is essential. This will help us avoid biases in data collection and analysis, ultimately leading to more effective strategies for preventing future zoonotic outbreaks.

Findings from the literature review show zoonotic diseases threaten both human health and wildlife conservation (Rahman et al. 2020; H. Li et al. 2021; González-Barrio 2022). Zoonoses can be transmitted in different ways, from direct contact with animals; indirect contact when animals and people share a contaminated area; transmission by vectors, such as ticks and insects; to consuming contaminated food or water (WHO 2020b). To reduce zoonotic transmission to humans, it is necessary to enhance our vigilance by building robust detection capabilities, and actively monitoring coronavirus presence in humans, wildlife, and livestock to inform human behaviors and minimize contact risks (Huong et al. 2020).

The reviewed literature emphasized the need for integrated One Health approaches to mitigate zoonotic disease risks and protect both human and wildlife health (Belay et al. 2017). This approach brings together human health, veterinary, and wildlife management sectors (WHO 2024). The One Health approach has been applied in Southeast Asia to address wildlife-related health risks. Examples include the Consultative Group on International Agricultural Research (CGIAR) Initiative on One Health, which focuses on tackling zoonotic disease risks in wildlife farming in Vietnam (CGIAR 2023). The Wildlife Conservation Society (WCS) also utilizes this approach to address challenges to biodiversity conservation and the increased risk of disease spillover arising from conditions along the wildlife trade chain (WCS 2020). Further research using the One Heath approach is crucial to strengthen disease surveillance and prevent future outbreaks (Nguyen et al. 2024). Studying how scientists, policymakers, and government implement this approach could be particularly valuable.

While our paper is comprehensive and systematic, we note several limitations. First, our restriction to English-language publications may have overlooked relevant studies published in other languages, introducing language bias. Second, variations in surveillance capabilities and laboratory methods across different countries may have influenced the comparability of pathogen detection rates, possibly affecting the reliability of our findings. Lastly, our inclusion of only published studies may have resulted in overrepresentation of studies with positive results; however, we made sure to include studies reporting null detection of pathogens. Overall, this study provides important insights into the range of wildlife zoonoses and future directions for wildlife zoonoses management based on experiences from Southeast Asia.

## 5. Conclusion

The identification, characterization, and monitoring of wildlife zoonotic pathogens is a growing area of research. This trend follows the recent interest in disease transmission from wildlife to humans since the 2020 outbreak of COVID-19. The reviewed publications highlight the importance of wildlife disease surveillance, particularly focusing on animals with frequent human contact. It is also crucial that this surveillance extends to individuals in close proximity to animals to better understand spillover mechanisms and transmission drivers. Concurrently, there is a need for further development initiatives aimed at enhancing hygiene practices, biosecurity, raising awareness about health risks associated with wildlife meat products, promoting alternative livelihoods, and advocating for wildlife conservation efforts to protect people from such risks. These endeavors should embrace a One Health approach, facilitating collaboration among wildlife, veterinary, medical, and conservation sectors, as well as across health and social science disciplines. Such collaboration is essential to ensure that these efforts are practical and effective. Finally, ongoing evaluation of these initiatives and the establishment of mechanisms for knowledge sharing are crucial to advancing zoonoses control efforts, recognizing that zoonotic diseases transcend geographical borders. Collectively, our findings offer an evidence base of wildlife zoonotic pathogens and their risk factors in Southeast Asia, serving as a valuable resource to guide future prevention efforts.

## Supplementary Material

Supplemental Material

## Data Availability

The authors confirm that the data supporting the findings of this study are available within the article [and/or] its supplementary materials.
